# Real-world evidence for secukinumab in UK patients with psoriatic arthritis or radiographic axial spondyloarthritis: interim 2-year analysis from SERENA

**DOI:** 10.1093/rap/rkad055

**Published:** 2023-08-21

**Authors:** Karl Gaffney, Nicola Gullick, Kirsten MacKay, Yusuf Patel, Raj Sengupta, Tom Sheeran, Louise Hemmings, Paula Pamies

**Affiliations:** Department of Rheumatology, Norfolk and Norwich University Hospitals, NHS Foundation Trust, Norwich, UK; University Hospital Coventry & Warwickshire, Warwick Medical School, University of Warwick, Coventry, UK; Rheumatology, Torbay and South Devon NHS Foundation Trust, Torquay, UK; Hull University Teaching Hospitals NHS Trust, Hull, UK; Royal National Hospital for Rheumatic Diseases, Royal United Hospitals, Bath, UK; University of Wolverhampton, Royal Wolverhampton NHS Trust, Wolverhampton, UK; Immunology, Novartis Pharmaceuticals UK Ltd, London, UK; Immunology, Novartis Pharmaceuticals UK Ltd, London, UK

**Keywords:** drug survival, PsA, radiographic axial spondyloarthritis, real-world evidence, secukinumab

## Abstract

**Objectives:**

The aim was to evaluate retention rates for secukinumab in patients with active PsA or radiographic axial spondyloarthritis (r-axSpA) treated in routine UK clinical practice.

**Methods:**

SERENA (CAIN457A3403) is an ongoing, non-interventional, international study of patients with moderate-to-severe chronic plaque psoriasis, active PsA or active r-axSpA, who had received secukinumab for ≥16 weeks before enrolment. The primary objective of this interim analysis was to assess treatment retention rates in patients with PsA or r-axSpA who were enrolled and followed for ≥2 years at centres in the UK. The safety analysis set includes all patients who received at least one dose of secukinumab. The target population set includes all patients who fulfilled the patient selection criteria.

**Results:**

The safety set comprised 189 patients (PsA, *n* = 81; r-axSpA, *n* = 108), and the target population set comprised 183 patients (PsA, *n* = 78; r-axSpA, *n* = 105). In the safety set, 107 patients (45 of 81 with PsA and 62 of 108 with r-axSpA) had previously received a biologic agent. Retention rates were similar between patients with PsA and r-axSpA after 1 year (PsA 91.0%, 95% CI: 84.0, 98.0; r-axSpA 89.2%, 95% CI: 82.7, 95.7) and 2 years (PsA 77.6%, 95% CI: 67.6, 87.7; r-axSpA 76.2%, 95% CI: 67.4, 85.0) of observation. Overall, 17.5% of patients (33 of 189) experienced at least one treatment-related adverse event, and 12.7% of patients (24 of 189) discontinued secukinumab because of adverse events.

**Conclusion:**

This analysis of real-world data from the UK demonstrates high retention rates for secukinumab over 2 years in patients with PsA or r-axSpA, with a favourable safety profile.

Key messages

- Few data exist regarding retention of secukinumab in UK patients with PsA/axial spondyloarthritis.
- In UK patients, retention rates were 90% and 77% after 1 and 2 years, respectively.
- The results demonstrate sustained effectiveness, good retention rates and a favourable safety profile for secukinumab.

## Introduction

PsA and axial spondyloarthritis (axSpA) are chronic autoimmune disorders that share common immunopathological and clinical features [1, 2]. PsA is characterized by inflammation of the axial and peripheral joints, entheses and skin [1, 2]. Extra-musculoskeletal manifestations can include uveitis and IBD [1]. PsA is associated with impaired physical function and poor quality of life (QoL) [3–5]. In the UK, the prevalence of PsA is estimated to be 0.19% [6].

AxSpA is characterized by involvement of the axial skeleton and SI joints [1, 2]. Changes in the SI joints can be detected by conventional radiography in patients with radiographic axSpA (r-axSpA), or by MRI alone in patients with non-radiographic axSpA (nr-axSpA) [1]. Clinical hallmarks include inflammatory back pain, enthesitis, stiffness and fatigue, which lead to functional impairment and reduced QoL [1, 3, 7]. Approximately one in three patients with axSpA have an extra-musculoskeletal manifestation [8]. The prevalence of axSpA in the UK is estimated to be 0.3% [9].

IL-17-mediated immune responses are important in the pathogenesis of PsA and axSpA, leading to joint inflammation, joint destruction and new bone formation [1, 2, 10, 11]. Secukinumab is a fully human anti-IL-17A mAb approved for the treatment of moderate-to-severe plaque psoriasis in patients aged ≥6 years, in adults with active PsA and an inadequate response to prior DMARD therapy, in adults with r-axSpA who have responded inadequately to conventional therapy, and in adults with nr-axSpA who have responded inadequately to NSAIDs and who have objective evidence of inflammation (i.e. via MRI or elevated CRP levels) [12]. The clinical trial programme for secukinumab has demonstrated sustained efficacy and a consistent safety profile in patients with psoriasis [13], PsA [14–18], r-axSpA [19–22] and nr-axSpA [23].

Evidence is now accruing on secukinumab for the treatment of PsA or r-axSpA in routine clinical practice [24–31]; however, little real-world evidence exists regarding the long-term retention of secukinumab in patients with PsA and r-axSpA in the UK. Therefore, we conducted an interim analysis of data from an ongoing real-world study to evaluate the retention, safety and effectiveness of secukinumab in patients with PsA or r-axSpA in the UK.

## Methods

SERENA (CAIN457A3403) is a non-interventional study in patients with moderate-to-severe chronic plaque psoriasis, active PsA or active r-axSpA, and is currently ongoing in 19 countries (Austria, Belgium, Bulgaria, Croatia, Czech Republic, Estonia, France, Germany, Greece, Hungary, Ireland, Israel, Italy, the Netherlands, Portugal, Russia, Slovenia, Switzerland and the UK) [32]. The study design, patient selection criteria and a comprehensive description of the outcome measures and data analysis plan have been published elsewhere [33]. All included patients provided written informed consent. This study was conducted in accordance with Good Clinical Practice and the Declaration of Helsinki of 1964 and its later amendments. The study was approved by the East of England Ethics committee. Eligible patients had active psoriasis, PsA or r-axSpA and were prescribed secukinumab by the treating physician. All patients should have received secukinumab for ≥16 weeks before enrolment [32]. Patients are to be observed for a minimum of 5 years or until secukinumab is discontinued.

Data collected include demographics, prior treatment for psoriasis, PsA or r-axSpA, and disease-specific effectiveness and QoL measures [32]. For patients with PsA, assessments include the 78 Tender Joint Count (TJC), 76 Swollen Joint Count (SJC) and dactylitis [34, 35], Leeds Enthesitis Index [36], Physician Global Assessment (PGA) [35], pain as measured by a 10 cm visual analogue scale (VAS) [37], HAQ—Disability Index (HAQ-DI) and Functional Assessment of Chronic Illness Therapy-Fatigue (FACIT-Fatigue) [35]. For patients with r-axSpA, assessments include BASDAI [38], CRP or high-sensitivity CRP (hsCRP) [39], Ankylosing Spondylitis DAS (ASDAS) [38], total spinal pain as measured by VAS (0–10 cm) and patient’s global assessment of disease activity [40]. Safety was assessed by reports of treatment-emergent adverse events (TEAEs), serious adverse events (SAEs) and adverse events (AEs) of special interest, defined as major adverse cardiac events (MACEs), *Candida* infections, IBD, uveitis and malignancy.

The primary objective of SERENA is to document the long-term retention of secukinumab in routine clinical practice [32]. Eligible patients were aged ≥18 years, with moderate-to-severe plaque psoriasis, PsA or AS, and had received secukinumab for ≥16 weeks before study enrolment. Patients with medical or psychological conditions that might prevent them from participating in the study and those participating in other clinical trials were excluded.

The safety analysis set includes all patients who received at least one dose of secukinumab after providing written informed consent, and the target population set includes all patients who fulfilled the inclusion/exclusion criteria. The analysis reported herein is restricted to the subset of patients with PsA or r-axSpA who were enrolled and followed for ≥2 years at centres in the UK. At the time of trial initiation, secukinumab was not approved in the UK for the treatment of nr-axSpA; therefore, all axSpA patients had r-axSpA.

## Results

In total, 194 patients (82 with PsA and 112 with r-axSpA) were enrolled at UK centres between 26 July 2017 and 12 October 2018. Five patients (one with PsA and four with r-axSpA) were excluded from the safety set because they did not receive secukinumab after providing informed consent. Thus, the safety set comprised 189 patients (81 with PsA and 108 with r-axSpA). In addition to the five individuals excluded from the safety set, six patients were excluded from the target analysis population (two patients with PsA who did not fulfil the patient selection criteria; one patient with PsA and two patients with r-axSpA who had protocol deviations; and one patient with r-axSpA who did not fulfil the selection criteria and had a protocol deviation). Thus, the target population set comprised 183 patients (78 with PsA and 105 with r-axSpA). At the time of the interim analysis, the mean age was 49 years, 61.4% of the target population were male, and the mean time since diagnosis of PsA or r-axSpA was 9.1 years and 11.2 years, respectively (Table 1).

**Table 1.** Patient characteristics at the time of enrolment (safety set)

Variable	PsA	r-axSpA	Total
	(*n* = 81)	(*n* = 108)	(*n* = 189)
Age, mean (s.d.), years^a^	50.4 (10.9)	47.9 (12.4)	49.0 (11.8)
Male, *n* (%)	43 (53.1)	73 (67.6)	116 (61.4)
White, *n* (%)	72 (88.9)	95 (88.0)	167 (88.4)
BMI, mean (s.d.), kg/m^2^	30.7 (5.7)	28.2 (5.9)	29.0 (5.9)
<18.5, *n* (%)	0	1 (1.5)	1 (1.0)
≥18.5 to <25, *n* (%)	4 (12.1)	19 (27.9)	23 (22.8)
≥25 to <30, *n* (%)	10 (30.3)	21 (30.9)	31 (30.7)
≥30, *n* (%)	19 (57.6)	27 (39.7)	46 (45.5)
Smoking status, *n* (%)^b^			
Current	15 (18.8)	25 (23.1)	40 (21.3)
Former	23 (28.8)	28 (25.9)	51 (27.1)
Never	28 (35.0)	39 (36.1)	67 (35.6)
Unknown	14 (17.5)	16 (14.8)	30 (16.0)
Time since disease diagnosis, mean (s.d.), years	9.1 (7.8)	11.2 (11.2)	–
Duration of secukinumab treatment prior to inclusion, mean (s.d.) [range], years	0.5 (0.3)	0.7 (0.4)	0.6 (0.3)
	[0.2, 1.4]	[0.1, 2.0]	[0.1, 2.0]

a For patients whose age was missing and only year of birth collected, age was calculated as 1 July in the year of birth.

b Proportions do not add up to 100% because data were missing for some patients.

r-axSpA: radiographic axial spondyloarthritis.

As per the protocol, all patients had been receiving secukinumab at the time of enrolment (baseline). The mean duration of treatment with secukinumab was 0.5 years in patients with PsA and 0.7 years in patients with r-axSpA (Table 1). At baseline, for patients with PsA, the mean (s.d.) SJC was 4.5 (6.0), the mean TJC was 12.8 (16.0), the prevalence of dactylitis was 11.9%, and the prevalence of enthesitis was 63.0%. At baseline, for patients with r-axSpA, the mean BASDAI score was 3.4 (2.7), mean VAS scores for nocturnal back pain and total back pain were 3.71 (2.94) and 3.52 (2.82), respectively, and the mean ASDAS-CRP score was 2.4 (1.1). At the time of enrolment, 2.6% of patients (5 of 189) had co-morbid uveitis (one patient with PsA and four patients with r-axSpA) and 1% of patients (2 of 189) had co-morbid IBD (one patient with PsA had Crohn’s disease and one patient with r-axSpA had ulcerative colitis).

### Treatment history and concomitant treatment

Of the 189 patients in the safety set, 82 (43.4%) were biologic naïve and 107 (56.6%) had received previous treatment with a biologic agent before initiation of secukinumab treatment (Table 2). Among patients with PsA, 55.6% (45 of 81) had previously received at least one biologic agent, 19.8% (16 of 81) had received two, and 18.5% (15 of 81) had received three or more before starting secukinumab. Of the 45 individuals who had received a biologic agent, 40 (88.9%) had discontinued because of lack of efficacy.

**Table 2.** Treatment received before and concomitant with secukinumab (safety set)

Treatment, *n* (%)	PsA	r-axSpA	Total
	(*n* = 81)	(*n* = 108)	(*n* = 189)
Prior treatment history with a biologic^a^^,^^b^			
Received ≥1 prior biologic	45 (55.6)	62 (57.4)	107 (56.6)
Received 1 prior biologic	14 (17.3)	33 (30.6)	47 (24.9)
Received 2 prior biologics	16 (19.8)	17 (15.7)	33 (17.5)
Received ≥3 prior biologics	15 (18.5)	12 (11.1)	27 (14.3)
Treatment regimens before secukinumab			
NSAID	5 (6.2)	17 (15.7)	22 (11.6)
DMARD	16 (19.8)	6 (5.6)	22 (11.6)
NSAID plus DMARD	6 (7.4)	1 (0.9)	7 (3.7)
Biologic (or biosimilar)	13 (16.0)	25 (23.1)	38 (20.1)
NSAID plus biologic (or biosimilar)	3 (3.7)	21 (19.4)	24 (12.7)
DMARD plus biologic (or biosimilar)	17 (21.0)	8 (7.4)	25 (13.2)
NSAID, DMARD plus biologic (or biosimilar)	12 (14.8)	8 (7.4)	20 (10.6)
Not documented/other	9 (11.1)	22 (20.4)	31 (16.4)
Treatment concomitant with secukinumab^c^^,^^d^			
NSAID	13 (16.0)	42 (38.9)	55 (29.1)
DMARD	24 (29.6)	12 (11.1)	36 (19.0)
NSAID plus DMARD	16 (19.8)	6 (5.6)	22 (11.6)
Biologic (or biosimilar)	1 (1.2)	1 (0.9)	2 (1.1)
NSAID plus biologic (or biosimilar)	0	2 (1.9)	2 (1.1)
DMARD plus biologic (or biosimilar)	0	1 (0.9)	1 (0.5)
Other	10 (12.3)	6 (5.6)	16 (8.5)
No documented treatment	17 (21.0)	38 (35.2)	55 (29.1)

a Among patients with PsA, prior biologic treatments included adalimumab (*n* = 31, 38.3%), etanercept (*n* = 25, 30.9%), golimumab (*n* = 16, 19.8%), ustekinumab (*n* = 15, 18.5%), infliximab (*n* = 6, 7.4%), certolizumab pegol (*n* = 5, 6.2%) and other biologic (*n* = 3, 3.7%).

b Among patients with AS, prior biologic treatments included adalimumab (*n* = 37, 34.3%), etanercept (*n* = 31, 28.7%), golimumab (*n* = 10, 9.3%), certolizumab pegol (*n* = 10, 9.3%), infliximab (*n* = 2, 1.9%) and other biologic (*n* = 4, 3.7%).

c Among patients with PsA, concomitant DMARD therapy included MTX (*n* = 28, 34.6%), SSZ (*n* = 10, 12.3%), LEF (*n* = 6, 7.4%), HCQ (*n* = 1, 1.2%) and CSA (*n* = 1, 1.2%).

d Among patients with r-axSpA, concomitant DMARD therapy included MTX (*n* = 11, 10.2%), SSZ (*n* = 9, 8.3%) and LEF (*n* = 2, 1.9%).

r-axSpA: radiographic axial spondyloarthritis.

Among patients with r-axSpA, 57.4% (62 of 108) had previously received at least one biologic agent, 15.7% (17 of 108) had received two agents, and 11.1% (12 of 108) had received three or more agents before starting secukinumab therapy. Of the 62 individuals who had received a biologic, 52 (83.9%) had discontinued biologics owing to lack of efficacy.

Of the patients with PsA who were receiving secukinumab, 35.8% (29 of 81) were receiving a concomitant NSAID and 63.0% (51 of 81) were receiving a concomitant DMARD alone or in combination with other agents (Table 2). In patients with r-axSpA who were receiving secukinumab, 46.3% (50 of 108) were receiving a concomitant NSAID and 21.3% (23 of 108) were receiving a concomitant DMARD alone or in combination with other agents (Table 2). A substantial minority of patients [21.0% (17 of 81) with PsA and 35.2% (38 of 108) with r-axSpA] were receiving no other documented treatment in combination with secukinumab.

### Main outcomes (target population set)

The recommended dose of secukinumab for patients with PsA and axSpA is 150 mg (300 mg in patients with PsA and concomitant moderate-to-severe plaque psoriasis or an inadequate response to a TNF-α inhibitor), with the possibility of increasing to 300 mg as needed. At baseline, most patients were receiving secukinumab at a dose of 150 mg (74.0%, 134 of 181); however, the modal dose at baseline differed by indication. In the subset of patients with PsA, more patients were receiving secukinumab 300 mg (56.6%, 43 of 76; dosing interval of 4 weeks) at baseline than 150 mg (baseline dose was missing for two patients), which is likely to reflect the proportion of patients with prior inadequate response to a TNF-α inhibitor. In contrast, in the subset of patients with r-axSpA, most patients (96.2%, 101 of 105) were receiving 150 mg at baseline, whereas only 3.8% (4 of 105) were receiving the 300 mg dose.

At the time of the interim analysis, secukinumab treatment was ongoing in 73.2% of patients (134 of 183) overall in the target analysis population; 78.2% of patients (61 of 78) with PsA and 69.5% of patients (73 of 105) with r-axSpA. Of the 17 patients with PsA who discontinued secukinumab, 9, 3 and 2 individuals, respectively, had discontinued treatment for lack of efficacy, physician decision and patient decision, while 3 patients discontinued for other reasons (Supplementary Table S1, available at *Rheumatology Advances in Practice* online). Likewise, of the 32 patients with r-axSpA who discontinued secukinumab, 11, 8 and 6 had discontinued treatment for the same three reasons, respectively, while 7 patients discontinued treatment for other reasons (Supplementary Table S1, available at *Rheumatology Advances in Practice* online).

**Figure 1.**
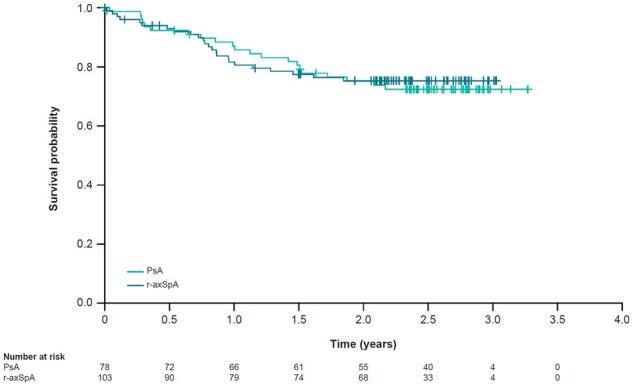
Time to treatment discontinuation. r-axSpA: radiographic axial spondyloarthritis

The mean time to treatment discontinuation was similar between patients with PsA and r-axSpA (Fig. 1). High retention rates were observed after 1 year (90.0%, 95% CI: 85.3, 94.7) and 2 years (76.8%, 95% CI: 70.3, 83.3) of treatment with secukinumab. Moreover, retention rates were similar in patients with PsA and r-axSpA after 1 year of observation (PsA 91.0%, 95% CI: 84.0, 0.98; r-axSpA 89.2%, 95% CI: 82.7, 95.7) and after 2 years of observation (PsA 77.6%, 95% CI: 67.6, 87.7; r-axSpA 76.2%, 95% CI: 67.4, 85.0).

**Figure 2.**
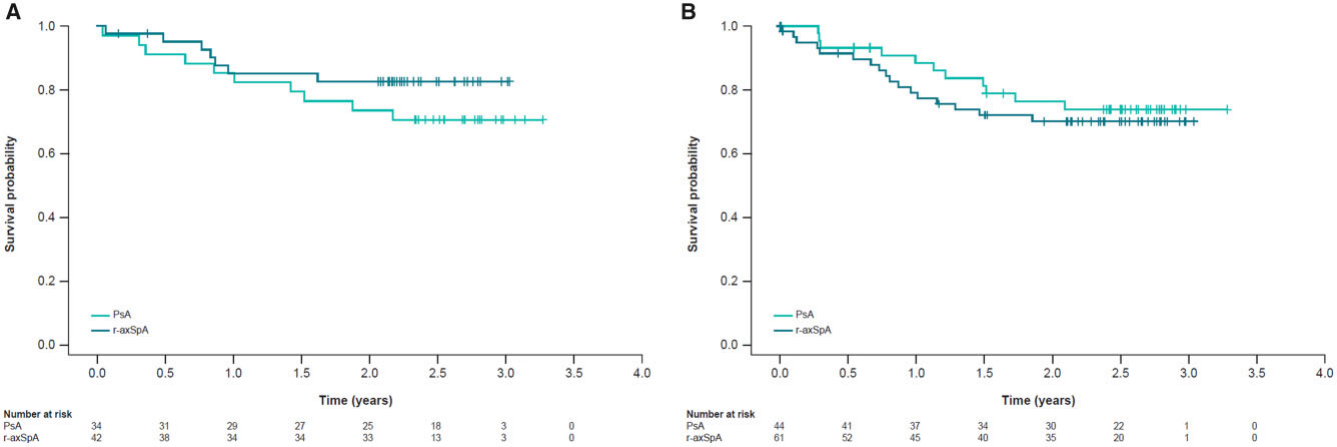
Time to treatment discontinuation by history of treatment with biologic agents. (A) Treatment naïve. (B) Biologic pre-treated. r-axSpA: radiographic axial spondyloarthritis

When patients were analysed according to their biologic treatment history, the mean time to discontinuation was similar between biologic-naïve and biologic pre-treated patients (Fig. 2). However, fewer treatment-naïve r-axSpA patients than treatment-naïve PsA patients discontinued treatment after 2 years of observation.

Treatment interruptions of >2 months were reported in 16 patients (5 of 78 with PsA and 11 of 105 with r-axSpA) after a mean (s.d.) treatment duration of 536 (301) days in patients with PsA and 408 (323) days in patients with r-axSpA. Reasons for treatment interruptions among patients with PsA included AEs (*n* = 3) and missing/other reasons (*n* = 2), and in patients with r-axSpA they included coronavirus disease 2019-related reasons (*n* = 2), patient decision (*n* = 1), treatment change according to product information (*n* = 1) and missing/other reasons (*n* = 7). The mean (s.d.) duration of these treatment interruptions was 157 (91) days in patients with PsA and 229 (226) days in patients with r-axSpA.

In patients with PsA, mean SJCs and TJCs remained stable throughout 2 years of treatment with secukinumab (Table 3). Overall, few patients had dactylitis or enthesitis, and the absolute number decreased over 2 years of treatment. Other efficacy outcomes remained stable throughout 2 years of observation, including total pain scores, the proportion of patients rated as 0/1 on the PGA, the proportion of patients with nail involvement and QoL measures (mean HAQ-DI and FACIT-Fatigue scores).

**Table 3.** Efficacy responses with secukinumab in PsA and radiographic axial spondyloarthritis cohort up to 2 years (target population set)

Endpoints	Enrolment	Year 1	Year 2
**PsA (*N* = 78)**			
Swollen joint count, mean ± s.d. (*M*)	4.5 ± 6.0 (36)	3.3 ± 4.6 (27)	3.6 ± 4.1 (14)
Tender joint count, mean ± s.d. (*M*)	12.8 ± 16.0 (36)	10.7 ± 11.3 (27)	9.2 ± 9.3 (14)
Presence of dactylitis, *n*/*M* (%)	8/67 (11.9)	3/52 (5.8)	3/35 (8.6)
Presence of enthesitis, *n*/*M* (%)	17/27 (63.0)	12/29 (41.4)	9/12 (75.0)
Total pain (VAS), mean ± s.d. (*M*)	38.9 ± 23.0 (39)	42.5 ± 26.6 (41)	56.1 ± 26.2 (18)
PGA 0/1 response, *n*/*M* (%)	14/24 (58.3)	19/32 (59.4)	9/11 (81.8)
Nail involvement, *n*/*M* (%)	9/65 (13.8)	4/50 (8.0)	5/36 (13.9)
HAQ-DI, mean ± s.d. (*M*)	1.1 ± 0.8 (35)	1.1 ± 0.7 (30)	1.1 ± 0.7 (11)
FACIT-Fatigue, mean ± s.d. (*M*)	29.8 ± 11.3 (32)	29.8 ± 12.1 (30)	30.5 ± 10.0 (11)
Total PASI score, mean ± s.d. (*M*)	1.5 ± 4.3 (24)	1.5 ± 2.3 (21)	0.6 ± 1.3 (9)
**r-axSpA (*N* = 105)**			
BASDAI, mean ± s.d. (*M*)	3.4 ± 2.7 (91)	2.9 ± 2.7 (46)	2.3 ± 2.9 (21)
Nocturnal back pain (VAS), mean ± s.d. (*M*)	37.1 ± 29.4 (60)	27.6 ± 29.3 (33)	16.3 ± 23.0 (12)
Total back pain (VAS), mean ± s.d. (*M*)	35.2 ± 28.2 (74)	31.9 ± 30.6 (48)	18.6 ± 26.7 (17)
CRP >5 *n*/*M* (%)	17/45 (37.8)	16/41 (39.0)	7/15 (46.7)
ASDAS-CRP mean ± s.d. (*M*)	2.4 ± 1.1 (28)	2.3 ± 1.1 (20)	2.4 ± 1.6 (8)
Patient’s global assessment of disease activity, mean ± s.d. (*M*)	5.3 ± 2.6 (64)	4.9 ± 2.8 (36)	4.8 ± 2.8 (16)
FACIT-Fatigue, mean ± s.d. (*M*)	27.3 ± 11.8 (59)	29.6 ± 9.6 (26)	30.7 ± 10.2 (10)

ASDAS: Ankylosing Spondylitis DAS; FACIT-Fatigue: Functional Assessment of Chronic Illness Therapy—Fatigue subscale score; HAQ-DI: HAQ—Disability Index; *M*: number of patients with evaluation; *n*: number of patients; *N*: number of patients in the study population; PASI: Psoriasis Area and Severity Index; PGA: Physician’s Global Assessment; r-axSpA: radiographic axial spondyloarthritis; VAS: visual analogue scale (0–100).

In patients with r-axSpA, mean BASDAI scores were sustained, and scores for total back pain and nocturnal back pain remained stable throughout 2 years of treatment with secukinumab (Table 3). Likewise, disease activity, as indicated by mean ASDAS-CRP values, remained stable throughout 2 years of observation.

### Safety (safety set)

Overall, 81.5% (66 of 81) and 53.7% (58 of 108) of patients with PsA and r-axSpA, respectively, experienced at least one TEAE, 21.0% (17 of 81) and 14.8% (16 of 108) of patients, respectively, experienced at least one treatment-related TEAE, and 11.1 (9 of 81) and 13.9% (15 of 108), respectively, discontinued treatment with secukinumab because of TEAEs (Table 4). Among patients with PsA, 15 SAEs were reported, with 13.6% of patients (11 of 81) experiencing at least one; among patients with r-axSpA, nine SAEs were reported, with 8.3% of patients (9 of 108) experiencing at least one. One death was reported during the observation period, which was considered unrelated to secukinumab treatment.

**Table 4.** Overall safety profile in PsA and r-axSpA patients (safety set)

Variable	PsA		r-axSpA	
	(*n* = 81)		(*n* = 108)	
Total exposure time, years	178.6		199.0	
Mean exposure time, years	2.2		1.8	
Patients with at least one TEAE, *n* (%)	66 (81.5)		58 (53.7)	
Patients with at least one treatment-related TEAE, *n* (%)^a^	17 (21.0)		16 (14.8)	
SAE^b^	15		9	
Patients with at least one SAE, *n* (%)	11 (13.6)		9 (8.3)	
Patients with TEAEs leading to death, *n* (%)	0		1 (0.9)^c^	
Patients with TEAEs leading to discontinuation, *n* (%)^c^	9 (11.1)		15 (13.9)	
**TEAEs of special interest**	***n* (%)**	**EAIR** ^d^	***n* (%)**	**EAIR** ^d^
*Candida* infections	1 (1.2)	0.5	1 (0.9)	0.5
Oral candidiasis	0	0	1 (0.9)	0.5
Malignancy	0	0	1 (0.9)	0.5
MACE	1 (1.2)	0.5	2 (1.9)	1.0
IBD	0	0	0	0
Uveitis	0	0	0	0

a AE/SAE with relationship ‘suspected’.

b Includes infections and infestations (*n* = 6: *Campylobacter* gastroenteritis, diverticulitis, lower respiratory infection, tooth abscess, tuberculosis, urosepsis); cardiac disorders [*n* = 3: acute myocardial infection (2), atrial fibrillation]; renal and urinary disorders (*n* = 3: nephrolithiasis, post-streptococcal glomerulonephritis, renal failure); nervous system disorders (*n* = 2: ataxia, ischaemic stroke); gastrointestinal disorders (*n* = 2: abdominal hernia, umbilical hernia); anaemia; cholecystitis; osteonecrosis; respiratory failure; psoriasis; metastatic malignant melanoma; death.

c Not related to treatment.

d Incidence rate per 100 patient-years calculated as: [total number of events in study interval/total patient (years in study interval^†^)] ×100. ^†^(Date of study completion or end date of study interval or date of discontinuation or interim cut-off date − date of informed consent + 1)/365.25.

EAIR: exposure-adjusted incidence rate; MACE: major adverse cardiac event; r-axSpA: radiographic axial spondyloarthritis; SAE: serious adverse event; TEAE: treatment-emergent adverse event.

The overall incidence of AEs of special interest was low in patients with PsA or r-axSpA (Table 4). Overall, three patients experienced MACEs, including ischaemic stroke in a 46-year-old patient with PsA who was a current smoker at study start, and acute myocardial infarction in two patients with r-axSpA (a 45-year-old patient who was a current smoker at study start and a 48-year-old patient who had never smoked). Two patients developed *Candida* infections. One patient with PsA receiving concomitant treatment with fluticasone developed mild laryngeal candidiasis, which prompted a temporary (6-week) interruption of secukinumab therapy. One patient with r-axSpA developed mild oral candidiasis, which resolved without any changes to the secukinumab treatment regimen. One patient with r-axSpA was diagnosed with a malignancy (malignant melanoma). No new cases or flares of IBD or uveitis were reported during the study.

## Discussion

A large number of international clinical trials have confirmed the safety and efficacy of secukinumab in patients with PsA or axSpA [25, 26, 27, 28, 29, 30, 41]. This interim analysis of UK data from the ongoing SERENA trial provides insight into how secukinumab is used and how patients respond in a UK health-care setting and facilitates comparison with other real-world data on secukinumab use. The National Institute for Health and Care Excellence (NICE) recommends secukinumab as an option for treating active PsA in patients with three or more swollen joints and who have not responded to at least two standard DMARDs or who have not responded to TNF-α inhibitors [42], and for treating active r-axSpA in adults whose condition has responded inadequately to conventional therapies [43]. The population included in the present study was treated in accordance with these recommendations, in that patients had generally received extensive treatment with NSAIDs, DMARDs and biologic therapies before initiating treatment with secukinumab; some biologic-experienced patients had received three or more biologics before starting secukinumab. Furthermore, NICE recommends that the response to treatment should be assessed after 16 weeks in patients with PsA or r-axSpA and continued only if there is clear evidence of response [42, 43]. The SERENA protocol specified that patients were eligible for enrolment after 16 weeks of treatment [32], meaning that the baseline data correspond to the point at which NICE recommends evaluating the response to treatment. This means that there is a high likelihood that only patients with a satisfactory response to treatment were enrolled.

High retention rates of ∼91% at 1 year and ∼77% at 2 years were observed in UK patients with PsA or r-axSpA in the present analysis. These results are similar to the overall SERENA cohort (*n* = 1004), in which retention rates were 85% and 86%, respectively, in patients with PsA and AS at 1 year, and 75% and 79%, respectively, at 2 years [33]. There are several differences between the UK cohort and the overall population (438 sites across Europe) that might contribute to the slightly better retention rates in the UK after 1 year of treatment. The duration of secukinumab treatment before inclusion was shorter in the UK subgroup, both in patients with PsA (0.5 *vs* 1.0 years in the overall population) and r-axSpA (0.7 *vs* 0.9 years in the overall population) [33]. Thus, there is a difference in the duration of treatment with secukinumab and the duration of observation between the UK cohort and the overall population. A lower proportion of patients in the UK cohort (56% with PsA and 57% with r-axSpA *vs* 67% and 63%, respectively) had received prior treatment with a biologic agent [33]. Conversely, a higher proportion of patients in the UK cohort *vs* the overall population were affected by obesity (46% *vs* 26%, respectively) at baseline [33], which has been associated with higher secukinumab treatment retention in women in one small observational study [44]. In addition, a higher proportion of patients in the UK cohort *vs* the overall population were current or former smokers (48% *vs* 38%), and a higher proportion of patients with PsA had dactylitis (12% *vs* 6%) or enthesitis (63% *vs* 19%) at baseline [33], the impact of which on treatment response is uncertain. Given that the baseline data represent results after ∼16 weeks of treatment, it is not possible to gauge the extent of improvement from the start of secukinumab treatment. However, the data show that responses obtained after ∼16 weeks of treatment were sustained over the next 2 years of treatment with secukinumab.

To provide a context for interpreting these data, prior observational studies in the UK have reported retention rates of 79% and 73% after the first and second years of treatment with a first TNF-α inhibitor in patients with PsA [45], and 83% after a first TNF-α inhibitor in patients with axSpA [46].

Data on drug survival of secukinumab are becoming available from other real-world studies in patients with PsA or AS and show that secukinumab is effective and has a high retention rate over time [27–30]. It should be noted that these results pertain to retention from the start of secukinumab treatment and therefore differ from the present study, in which observation began after 16 weeks of treatment; thus, the reported retention rates are not comparable. Analysis of data from >2000 patients with PsA treated with secukinumab in 13 clinical practice registries participating in the European Spondyloarthritis Research Collaboration Network showed that secukinumab treatment was retained by 86% of patients after 6 months and by 76% after 12 months [28]. Higher retention rates were observed in biologic/targeted synthetic DMARD-naïve patients than in patients who had received two or more prior biologic/targeted synthetic DMARD agents at 6 months (90% *vs* 85%, respectively) and 12 months (82% *vs* 72%) [28], which shows that more extensive prior treatment is associated with somewhat lower response rates.

A nationwide cohort study (*n* = 2551) showed that, among Danish patients with PsA who received treatment with biologics and immunomodulators, drug survival rates were higher for agents that target IL-17, including secukinumab, alongside agents that target TNF and IL-12/23 [29]. The same analysis showed that, in patients with AS, drug survival by secukinumab was among the highest, both overall and in biologic-experienced patients in particular [29]. Likewise, among French patients with PsA (*n* = 6531), treatment with an agent targeting IL-17 (individual agents were not specified) was associated with higher persistence than treatment with agents targeting TNF or IL-12/23 [31].

Despite the Phase 3 EXCEED study showing greater secukinumab retention rates *vs* adalimumab when administered as first-line treatment [47], analyses of data collected in Nordic countries between 2015 and 2018 showed that there was no significant difference in treatment retention rates between adalimumab and secukinumab when used to treat patients with PsA [27] or as first- or second-line therapy for SpA [30]. That said, secukinumab was rarely used as first-line treatment [27].

Efficacy was not the primary objective of SERENA, and efficacy results are missing for many patients. However, among patients with PsA for whom efficacy data are available, the mean TJCs (4.5 ± 6.0) and SJCs (12.8 ± 16.0) at enrolment (∼6 months after starting treatment with secukinumab) are relatively high. This suggests that the disease must have been severe at baseline. Although it is not possible to comment on the extent of improvement between the start of treatment with secukinumab and the time of enrolment in SERENA, these data suggest that the patients started off with severe inflammation and that the degree of improvement after ∼6 months provided encouragement to continue treatment. In contrast, among patients with r-axSpA who provided data, the mean BASDAI score (3.4 ± 2.7) suggests that the disease was well controlled at enrolment and was in remission after 1 and 2 years.

In the SERENA UK cohort, treatment interruptions occurred infrequently and were attributed to diverse reasons, although it should be noted that AEs were not the most common reason for treatment interruptions. The most common reasons for discontinuation of secukinumab were lack of efficacy, physician decision and patient decision, whereas AEs were cited by few patients, which suggests that treatment was generally well tolerated. This finding is consistent with those of the overall SERENA study, in which the major reasons for drug discontinuation were lack of efficacy and patient decision [33]. The safety profile of secukinumab in the present study is consistent with that observed in large Phase 3 studies in patients with PsA [14–18] or r-axSpA [19, 22]. No new safety signals were detected; the incidence of TEAEs of special interest was low, and no new cases of uveitis or IBD were reported. This is consistent with analyses of data from Phase 3 clinical trials suggesting that there is a low risk of uveitis or IBD with secukinumab treatment [48, 49].

Limitations of this real-world analysis have been described in detail elsewhere [32]. The most pertinent of these include the observational study design, lack of a control group, limited or missing data, and the small number of patients in the present analysis because it was restricted to one country. Given that patients were enrolled after receiving 16 weeks of treatment with secukinumab, the study is also subject to selection bias, because only patients with an initial response to secukinumab were included. Thus, the findings apply only to patients who are eligible to continue secukinumab treatment after having completed 16 weeks of initial treatment.

In conclusion, this analysis of real-world data from the UK demonstrates high retention rates and sustained efficacy of secukinumab over >2 years in patients with PsA or r-axSpA, consistent with the overall results of SERENA and with emerging data from other real-world studies. The overall incidence of TEAEs and TEAEs of special interest was low. The safety profile is consistent with that reported in clinical trials in patients with PsA or r-axSpA, and no new safety signals were observed.

## Supplementary Material

rkad055_Supplementary_Data

## Data Availability

Data are available upon reasonable request. All data relevant to the study are included in the article or uploaded as Supplementary Material (available at *Rheumatology Advances in Practice* online). The data sets generated and/or analysed during the current study are not publicly available. Novartis is committed to sharing with qualified external researchers’ access to patient-level data and supporting clinical documents from eligible studies. These requests are reviewed and approved on the basis of scientific merit. All data provided are anonymized to respect the privacy of patients who have participated in the study in line with applicable laws and regulations. The data may be requested by writing to the corresponding author.
