# Supplementary material for: Real-world evidence for secukinumab in UK patients with psoriatic arthritis or radiographic axial spondyloarthritis: interim 2-year analysis from SERENA
Source: Rheumatol Adv Pract. 2023 Aug 21;7(3):rkad055. doi: 10.1093/rap/rkad055 (PMC10472087; doi:10.1093/rap/rkad055)
Supplement: rkad055_Supplementary_Data [file rkad055_supplementary_data.docx]

**Supplementary** **Table S1. Reasons for discontinuing secukinumab treatment (target population set)**

| Reason for discontinuing treatment | Patients discontinuing treatment, n (%) | | |
| --- | --- | --- | --- |
|  | PsA  (n=17) | r-axSpA  (n=32) | Total  (N=49) |
| Lack of efficacy | 9 (52.9) | 11 (34.4) | 20 (40.8) |
| Physician decision | 3 (17.6) | 8 (25.0) | 11 (22.4) |
| Patient decision | 2 (11.8) | 6 (18.8) | 8 (16.3) |
| Death | 0 | 1 (3.1) | 1 (2.0) |
| Adverse event | 0 | 4 (12.5) | 4 (8.2) |
| Lost to follow-up | 2 (11.8) | 1 (3.1) | 3 (6.1) |
| Withdrawal of informed consent | 0 | 1 (3.1) | 1 (2.0) |
| Administrative reason | 1 (5.9) | 0 | 1 (2.0) |

PsA, psoriatic arthritis; r-axSpA, radiographic axial spondyloarthritis.
